# Cochrane methods - twenty years experience in developing systematic review methods

**DOI:** 10.1186/2046-4053-2-76

**Published:** 2013-09-20

**Authors:** Jackie Chandler, Sally Hopewell

**Affiliations:** 1 The Cochrane Collaboration, Oxford, UK; 2 The Cochrane Collaboration, London, UK; 3 INSERM, Paris U738, France; 4 The French Cochrane Centre, Paris, France; 5 Centre for Statistics in Medicine, University of Oxford, Oxford, UK

## Abstract

This year, The Cochrane Collaboration reached its 20th anniversary. It has played a pivotal role in the scientific development of systematic reviewing and in the development of review methods to synthesize research evidence, primarily from randomized trials, to answer questions about the effects of healthcare interventions. We introduce a series of articles, which form this special issue describing the development of systematic review methods within The Cochrane Collaboration. We also discuss the impact of Cochrane Review methods, and acknowledge the breadth and depth of methods development within The Cochrane Collaboration as part of the wider context of evidence synthesis. We conclude by considering the future development of methods for Cochrane Reviews.

## Archie Cochrane’s vision of the future in 1972

‘(The pathologist)..will be replaced by the medical scientist who will measure the effectiveness and efficiency of therapy in the hospital and the community and in conjunction with social scientists to assess the adequacy of community care...........I hope clinicians in the future will abandon the pursuit of the “margin of the impossible” and settle for “reasonable probability”.’ AL Cochrane (1972) *Effectiveness and Efficiency: Random Reflections on Health Services* The Nuffield Provincial Hospitals Trust [1].

## Introduction

The Cochrane Collaboration, a research synthesis organization, is celebrating its 20th Anniversary in 2013. Whilst taking stock of its achievements, it is identifying areas for improvement and development as pressures continue for the synthesis of the proliferation of research, in order to aid healthcare decision making [2]. Key to the development of the rigorous review model established by The Cochrane Collaboration is the methodological work that has been undertaken alongside review production. This has resulted in the formation of sixteen international methods specific networks developing methods for application in Cochrane Reviews.

## Background

Archie Cochrane, director of the MRC Epidemiology Unit in Cardiff, Wales, UK, at the time of writing the above evaluation of the National Health Service (UK), clearly articulated the need for applied scientific evidence over the expert opinion of clinicians [1]. In 1979 he suggested that ‘It is surely a great criticism of our profession that we have not organized a critical summary, by specialty or subspecialty, adapted periodically of all relevant randomized controlled trials’ [2]. This spurred Sir Iain Chalmers [3] and others to take the steps needed to set up, in 1993, The Cochrane Collaboration, which began with a meeting of 77 people from nine different countries. Since then, The Cochrane Collaboration has grown substantially. It is now an international network of more than 28,000 voluntary contributors from over 120 countries [4], which publishes full systematic reviews and their protocols in *The Cochrane Database of Systematic Reviews* (CDSR). This has published reviews online since 1996, via *The Cochrane Library.* The CDSR currently includes more than 2,300 review protocols and more than 5,600 full reviews have been published. The *Library* also contains, amongst other databases CENTRAL, the world’s largest repository of records for randomized trials, with more than 700,000 as of September 2013.

The Cochrane Collaboration has been at the forefront of systematic review methodology, pioneering a rigorous approach that standardizes a highly structured systematic review model. Key elements of the model are transparency and reproducibility of research methods. These include title registration, publication of a protocol and periodic updating of the subsequent published systematic review. The Cochrane Collaboration has drawn in an international spectrum of individuals to support and develop the methods for systematic reviews over this time. This article provides an overview of this work and the major contributions that The Cochrane Collaboration has made to systematic review methodology over the last 20 years. We introduce a series of articles that present and discuss key methodological developments within Cochrane, such as the development of the ‘Risk of bias’ tool [5] and ‘Summary of findings’ tables [6] which are now regularly being seen in systematic reviews. The articles also scan the methodological horizon to identify important developments for future Cochrane Reviews and systematic review methods more generally.

In recent years, The Cochrane Collaboration has broadened its original scope from the effects of interventions to address other types of uncertainty, with the appearance of systematic reviews of diagnostic test accuracy and a pilot project to examine the feasibility of reviews of prognosis. Methodological developments have also supported the appropriate use of non-randomized designs to assess adverse effects of interventions, and the enhancement of intervention reviews with brief economic summaries. Other developments include the introduction of statistical concepts such as network meta-analysis to allow indirect comparisons of multiple interventions. The Cochrane Collaboration is also developing expertise around the handling of intervention complexity in Cochrane Reviews, as well as incorporating qualitative evidence syntheses, to provide additional explanatory data on healthcare interventions.

The series of articles in this special issue highlights the current and future contribution of The Cochrane Collaboration to the overall quality, standing and rigor of systematic review methodology in the wider international community. It also highlights the contribution of Cochrane methods and the Cochrane Review model to the scientific discipline of systematic reviews.

A core component of methods development within The Cochrane Collaboration is the international methods specific networks referred to as Methods Groups. These groups are unique within the global evidence synthesis community, providing a rich resource of committed experts in systematic review methodology working across many disciplines. The flourishing of these groups over the years is indicative of an emergent academic discipline recognizing the requirement for robust methods to synthesize evidence for healthcare. These groups are responsible for contributing to the chapters in the *Cochrane Handbook of Systematic Reviews of Interventions*[7]. The Groups specialize in particular aspects of systematic review methodology, including: searching for studies, statistics and meta-analysis (MA) including prospective MA and individual participant data MA, assessing bias, use of non-randomized designs, the incorporation of qualitative and economic data, the applicability and interpretation of the findings of systematic reviews, patient reported outcomes, equity issues, screening and diagnostic tests, and prognosis. More recently, Methods Groups have formed specializing in the methods of indirect comparisons and network meta-analysis, and in the setting of priorities for systematic reviews and other research.

Since The Cochrane Collaboration was established two decades ago, the task of preparing and maintaining systematic reviews for a range of health and social topics, utilizing a wide range of approaches has expanded considerably. There are now several examples of other organizations producing evidenced based synthesis, including the Agency for Healthcare Research and Quality (ARHQ) in the US and the Joanna Briggs Institute based in Australia. The Campbell Collaboration, which works in partnership with The Cochrane Collaboration, produces systematic reviews on the effects of interventions in crime and justice, education, international development, and social welfare. The Centre for Reviews and Dissemination (CRD) part of the National Institute of Health Research (NIHR) in the UK, is one of a number of producers of systematic reviews and health technology assessments for the NIHR, but has also developed the international prospective register of systematic reviews (PROSPERO) [8] and continues its work to produce *The Database of Abstracts of Reviews of Effects*. Also based in the UK is the Evidence for Policy and Practice Information and Co-ordinating Centre that produces systematic reviews and develops review methods in social science and public policy. The Cochrane Collaboration has many ties with these organizations and members that work across these different agencies. It also has partnerships with organizations which cite, use or collaborate on systematic reviews and other initiatives. These include, for example, the World Health Organization [9], guideline developers such as the National Institute of Health and Clinical Excellence (NICE) in the UK [10] and the Guideline International Network (G-I-N), as well as other national agencies such as the Institute of Medicine in the US.

### The impact of 20 years of Cochrane methodology

Cochrane Reviews have made valuable contributions to healthcare research, practice and policy across a wide range of topics, for example by furthering our knowledge of falls prevention in older people, stroke, tobacco addiction, preventing and treating childhood obesity, and managing chronic diseases such as diabetes and emphysema. Not only have Cochrane Reviews established the role of some interventions in the management of health problems, they have also challenged the place of others. There is an expectation that new research should be informed by previous research, and, for example, *The Lancet* now asks authors to report the results of new research within the context of existing systematic review evidence [11].

However, quantifying the influence of Cochrane Reviews and methods is not easy. One crude metric is the impact factor of the CDSR, which is 5.785, ranking it 11th of 151 journals in the Medicine, General and Internal category. A recent evaluation by Shen and colleagues of the production and utilization of Cochrane Reviews, showed the rapid growth in the average annual output of reviews and the high citation rate of Cochrane Reviews in high income countries (for example, England, Australia, Canada and USA). However, they also identified the lack of production and the under utilization of Cochrane Reviews in other parts of the world, and proposed more applicable evidence production such as public health reviews [12]. The Cochrane Collaboration has recently established a satellite for its Public Health Review Group in India. The use of Cochrane evidence in clinical guidelines and other evidence-based recommendations is another guide to the impact of The Cochrane Collaboration. A recent study found that, as of July 2013, 1,158 Cochrane Reviews from 47 Cochrane Review Groups have been used to inform 238 clinical guidelines and other evidence-based recommendations from the Scottish Intercollegiate Guidelines Network (24 reviews), National Institute of Health and Care Excellence guidelines (115 reviews), and the World Health Organization (99 reviews) [13].

More specifically for impact of The Cochrane Collaboration on the methods of systematic reviews, the *Cochrane Handbook for Systematic Reviews of Interventions* has been cited over 6,600 times [14]. An annual journal supplement to *The Cochrane Library* (http://www.thecochranelibrary.com/view/0/CochraneMethods.html) keeps members of The Cochrane Collaboration apprised of the methodological work being undertaken within Cochrane, as well as commenting on other relevant published methodological work. Furthermore, Table 1 highlights some key contributions members of Cochrane Methods Groups have made to Cochrane Reviews. These include major methodological developments such as the early development of the *Cochrane Handbook*, bias assessment, the quantification of heterogeneity to measure the degree of inconsistency in the primary studies [15] and the more recent introduction of the ‘Summary of findings’ table based on GRADE considerations [16]. Additional historical information of the development of The Cochrane Collaboration and its Methods Groups can be found on The Cochrane Collaborations website (http://www.cochrane.org/about-us/history), and the annual Cochrane Colloquium is an important scientific platform, with oral and poster presentations showing the breadth of scientific developments in systematic review methodology [17].

**Table 1:** Key methodological developments in Cochrane Reviews

2014	‘Risk of bias’ tool extension for non standard randomized studies (for example, crossover and cluster trials) and non-randomized studies
2012	Introduction of the Methodological Expectations of Cochrane Intervention Reviews (MECIR) standards
2011	Launch of the Cochrane Methods Innovation fund
2008	Release of version 5 of RevMan incorporating ‘Risk of Bias’ tool
	Grade profiler software (GRADEpro) introduced for ‘Summary of findings’ tables in RevMan
2002	I^2^ statistics measuring inconsistency in meta-analysis [22]
1996	Launch of *The Cochrane Library* launched by Update Software incorporating *The Cochrane Database of Systematic Reviews*, *The Database of Abstracts of Reviews of Effectiveness, The Cochrane Controlled Trials Register* and *The Cochrane Review Methodology Database*
	Bias assessment classification system introduced for allocation concealment [27]
1994	First publication demonstration of *The Cochrane Database of Systematic Reviews*
	Publication of the first edition of the *Cochrane Handbook*[26]
	Registration of the first Methods Groups: Statistical MG and Individual Patient Data MG
1993	Formal launch of the Cochrane Collaboration at the first Cochrane Colloquium in Oxford, UK
	Release of version 1 of Review Manager (RevMan)
1992	Formal launch of the first UK Cochrane Centre in Oxford
1988	Publication of the first in a series of overviews (meta-analyses) in the *British Journal of Obstetrics and Gynaecology*
1976	Term ‘meta-analysis’ first introduced [25]
1972	Publication of Archie Cochrane’s *Effectiveness and Efficiency: Random Reflections on Health Services* which first drew attention to the collective ignorance about the effects of health care [1]

A notable recent example of impact of both new methodology and the Cochrane Review comes from the work of the Cochrane Acute Respiratory Infections Group and others on the Cochrane Review of neuraminidase inhibitors (including Tamiflu) for influenza [18]. This illustrates the challenges of implementing standard methodological expectations when synthesizing data from a substantial unpublished evidence base. By abandoning journal articles in favor of full technical reports as their primary sources of data, the authors have had to capture information from thousands of pages of information in order to assess and collect the outcome data they need for their analyses. Set in the context of conflicting national regulatory and drug licensing processes, the review provides a reference point for how systematic reviews may need to draw on new types of evidence, and the methodological tools that will be needed to do this in years to come.

### Article series

The articles in this special issue of *Systematic Reviews* illustrate the overall development and impact of research evidence synthesis in The Cochrane Collaboration over the last 20 years. The series begins with a personal reflection from Andy Oxman, who was instrumental in the early development of methodology in The Cochrane Collaboration and set out challenges for Cochrane [19] over ten years ago. In reflecting and updating these challenges and how they might be met, he notes that the Collaboration has come a long way, but that ‘a huge amount of work remains to be done’. He suggests a need to broaden the review structure to address different types of review questions, a wider use of non-randomized studies, the comparison of multiple interventions and the development of efficient updating strategies.

A collection of articles then focuses on principal methods and underpinning principles of systematic reviews, beginning with the identification and retrieval of studies where new developments in information are already leading to substantial changes. These include new techniques in semantic analysis, text mining and data linkage that identify the ‘meaning’ as oppose to just the ‘presence’ of the term, as well as developments in identifying unpublished data. Another article discusses the assessment of study bias and the development of the Cochrane ‘Risk of bias’ tool in 2008. This tool is being revised to improve its utility and reliability and extensions will support the assessment of bias in other types of research. In regard to meta-analysis methods, statistical inference and the presentation of the findings of reviews, articles from the Statistical Methods Group and the Applicability and Recommendations Methods Group show substantial developments in, for example, assessing statistical heterogeneity and the use of GRADE criteria to produce ‘Summary of findings’ tables.

Other articles in this series illustrate the diversity of evidence that is being incorporated into Cochrane Reviews. One of these discusses the introduction of reviews of diagnostic test accuracy (rather than the effects on health outcomes of particular tests), which have started to pave the way for Cochrane to include other types of systematic review alongside its more traditional reviews of the effects of interventions. The other articles focus on the development and implementation of methods to assess ‘cost’ in systematic reviews, which are being developed by the Campbell and Cochrane Economic Methods Group, and the challenges of incorporating qualitative research to enhance understanding of the wider context of healthcare interventions.

Across The Cochrane Collaboration, other specific methods networks are examining the appraisal and reporting of adverse effects, the use of non-randomized designs including the assessment of bias in these studies, the development of methods to prioritize and update reviews, and the consideration of equity in both the conduct and the interpretation of reviews.

### The future of methods development in Cochrane

The ongoing drive for The Cochrane Collaboration is to ensure that it produces high quality, relevant and up to date systematic reviews. In keeping with the need to continue to improve the quality of reporting [20-23], The Cochrane Collaboration has produced a set of standards. These standards [24] cover the conduct and reporting of reviews, including the reporting of protocols and the updating of reviews.

The Cochrane Collaboration is committed to pioneering research that will lead to further improvements in the methods used for Cochrane Reviews. As an example, the Cochrane Methods Innovation Fund has supported six methods related projects since 2012. These projects are investigating priority topics for Cochrane and include: methods of searching for unpublished trials, extensions to the Cochrane ‘Risk of bias’ tool to assess risk of bias in randomized trials with non-parallel-group designs and non-randomized studies, enhancing the acceptance and implementation of ‘Summary of findings’ tables, the assessment of complex interventions, addressing missing trial participant data and methods (network meta-analysis) for comparing multiple interventions.

A fundamental feature of Cochrane Reviews since the start of the Collaboration has been the requirement to update them periodically. Twenty years on, methodology research is seeking to establish the longevity of the clinical relevance of Cochrane Reviews, taking account of changes in methodology as well as additional evidence. This will help to ensure that the quality of each Cochrane Review improves alongside the incorporation of more recent studies [25]. This is a fundamental area of policy, and methodological development, not least because nearly 70% of Cochrane Reviews had not been updated in the last two years in 2012 [26]. Future research is needed to determine the feasibility and efficiency of updating and prioritization strategies, including statistical techniques [27,28], so that the necessary guidance can be prepared.

The need for systematic reviews to inform decision making in health and social care will remain into the foreseeable future [29]. This highlights the ongoing importance of the collaborative effort of The Cochrane Collaboration amongst others to continue to use sound methods to aggregate the ever-increasing number of new studies. Technological as well as methodological progress is key to advancing the aggregation and dissemination of systematic review evidence [30]. Some challenges for systematic reviews arise from successes in improving access to the potentially eligible studies including prospective registration of randomized trials [31], the ongoing push for greater availability of published as well as unpublished study reports [32] and the need to update reviews to inform and interpret new research will require robust methods, and resources. The capacity to identify and appraise the underlying research and to systematically synthesize the evidence will continue to be challenging for organizations such as The Cochrane Collaboration.

## Conclusion

This article outlined the breadth and diversity of systematic review methods that are being incorporated in Cochrane Reviews. These, and other, systematic reviews need to encompass a range of evidential data [33], to determine treatment effectiveness and to explain, inform, contextualize and triangulate the findings. Thus it seems appropriate in this 20th year of systematic review development that The Cochrane Collaboration’s contribution to this emergent academic discipline [34] is established.

## Abbreviations

I2: A statistic that quantifies inconsistency (heterogeneity) across studies in a systematic review; MECIR: Methodological Expectations of Cochrane Intervention Reviews; RevMan: Review management software, designed specifically for Cochrane Reviews; GRADE: The Grading of Recommendations, Assessments and Development and Evaluation; GRADEpro: GRADE profiler software to create ‘Summary of findings’ tables.

## Competing interests

JC and SH are employees of The Cochrane Collaboration or Cochrane Groups.

## Authors’ contributions

JC conceived the paper as part of an outline for this *Systematic Reviews* series and developed the initial draft. SH commented on and edited subsequent drafts of the manuscript. Both authors read approved the final manuscript.
